# Developmental Defects in Mutants of the PsbP Domain Protein 5 in *Arabidopsis thaliana*

**DOI:** 10.1371/journal.pone.0028624

**Published:** 2011-12-09

**Authors:** Johnna L. Roose, Laurie K. Frankel, Terry M. Bricker

**Affiliations:** Department of Biological Sciences, Biochemistry and Molecular Biology Section, Louisiana State University, Baton Rouge, Louisiana, United States of America; Ohio State University, United States of America

## Abstract

Plants contain an extensive family of PsbP-related proteins termed PsbP-like (PPL) and PsbP domain (PPD) proteins, which are localized to the thylakoid lumen. The founding member of this family, PsbP, is an established component of the Photosystem II (PS II) enzyme, and the PPL proteins have also been functionally linked to other photosynthetic processes. However, the functions of the remaining seven PPD proteins are unknown. To elucidate the function of the PPD5 protein (At5g11450) in Arabidopsis, we have characterized a mutant T-DNA insertion line (SALK_061118) as well as several RNAi lines designed to suppress the expression of this gene. The functions of the photosynthetic electron transfer reactions are largely unaltered in the *ppd5* mutants, except for a modest though significant decrease in NADPH dehydrogenase (NDH) activity. Interestingly, these mutants show striking plant developmental and morphological defects. Relative to the wild-type Col-0 plants, the *ppd5* mutants exhibit both increased lateral root branching and defects associated with axillary bud formation. These defects include the formation of additional rosettes originating from axils at the base of the plant as well as aerial rosettes formed at the axils of the first few nodes of the shoot. The root-branching phenotype is chemically complemented by treatment with the synthetic strigolactone, GR24. We propose that the developmental defects observed in the *ppd5* mutants are related to a deficiency in strigolactone biosynthesis.

## Introduction

The thylakoid membranes of plant chloroplasts contain the enzymes responsible for the photosynthetic reactions that convert solar energy into biochemical energy and molecular oxygen. These enzymes are the protein complexes Photosystem II (PS II), cytochrome *b_6_f*, Photosystem I (PS I), and ATP synthase. In addition, the thylakoid membranes also contain light-harvesting antenna complexes (LHCI and LHCII), chaperones for membrane protein integration, translocation complexes for targeting of lumenal proteins, NADPH dehydrogenase complexes (NDH), and a plastid terminal oxidase (PTOX) [1]. These molecular machines, in concert with an extensive network of regulators and factors required for biogenesis, collectively maintain optimal electron transfer. PS II performs the light-driven conversion of water to molecular oxygen. This multi-subunit membrane protein complex contains more than twenty protein subunits and numerous cofactors. The PsbP protein is an extrinsic protein associated with the lumenal face of the PS II enzyme near the oxygen-evolving complex (OEC). Biochemical reconstitution experiments have shown that PsbP is required for optimal oxygen-evolving activity at physiological calcium and chloride concentrations [2], [3], [4]. Moreover, PsbP is essential for autotrophy in both land plants [5], [6] and green algae [7], [8] and is required for normal thylakoid architecture in Arabidopsis [9].

The PsbP protein is the founding member of an extensive family of PsbP-related proteins which are also predicted to be localized to the thylakoid lumen of plant chloroplasts [3], [10], [11]. The PsbP protein is a well-characterized PS II subunit. The PsbP-like (PPL) proteins are more similar in sequence to the cyanobacterial PsbP protein homolog. Finally, the PsbP-domain (PPD) proteins are more divergent from the PsbP component than the PPL proteins [10]. In Arabidopsis, there are two *PSBP* genes, two *PPL* genes and seven *PPD* genes [10], [11]. Notably, the *PPL* and *PPD* genes are not just *PSBP* variants found within Arabidopsis, since corresponding homologs exist in all other plant genomes analyzed to date [11]. Additionally, the PPL and PPD proteins cannot complement the phenotype observed upon the genetic deletion of the PsbP protein [5], [6]. Proteomic studies in Arabidopsis have demonstrated that the majority of these PsbP family members exist as proteins within the thylakoid lumen [12], [13], [14], [15].

Initial genetic analysis of the PPL proteins using T-DNA insertion lines of Arabidopsis indicated that they contribute to photosynthetic function in a variety of ways. While the *ppl1* and *ppl2* mutants displayed growth rates similar to wild-type plants under standard laboratory growth conditions, both displayed defects in photosynthetic electron transfer [10]. The PPL1 protein has been implicated in facilitating PS II repair upon photodamage because the *ppl1* mutant plants were sensitive to high light treatment and displayed slower recovery from photoinhibition. The *ppl2* mutant plants had significantly decreased amounts of the NDH subunits, indicating that the PPL2 protein contributes to NDH complex accumulation and function. This result is interesting because it implies that the PPL2 protein has a function separate from PS II, but still contributes to electron transfer in the thylakoid membranes via the NDH complex and cyclic electron flow around PS I. A recent report comparing the gene co-regulation networks of the different PPL and PPD proteins predicted that the different protein family members could be classified as having either stress-related or NDH-related functions [16]. Members predicted to be part of the stress-responsive group include PPL1, PPD1, PPD3, PPD5 and PPD6. PPL2 and PPD2 were classified in the NDH-related group. While experimental data support this classification scheme for the PPL proteins, no experimental evidence to support the classifications of the PPD proteins is currently available. Here we report the characterization of Arabidopsis mutants of the PPD5 protein, examining their capacity for photosynthetic electron transport (linear and cyclic) and describing striking developmental and/or morphological defects observed in these mutant plant lines.

## Materials and Methods

### Plant Materials and Growth Conditions

**Figure 1:**
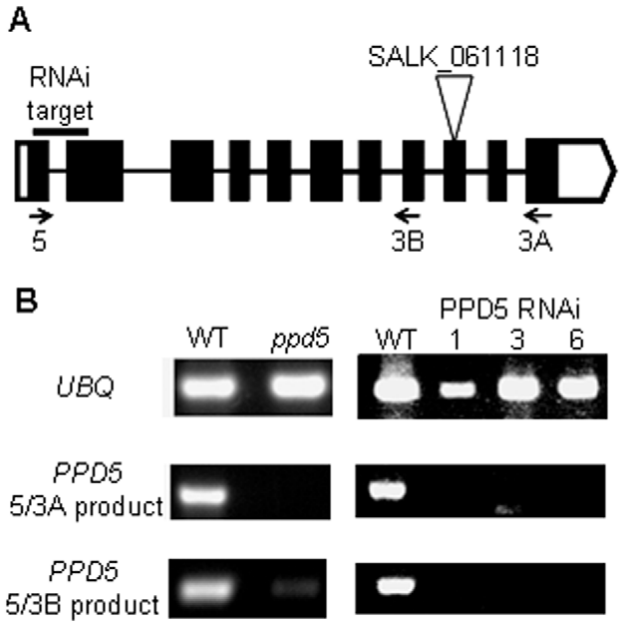
*PPD5* gene structure and expression analysis. (A) *PPD5* gene structure. The locations of the RNAi target sequence, the T-DNA insertion and primers used for expression analysis are shown. (B) RT-PCR amplification of *UBQ* and *PPD5* transcripts from WT, *ppd5*, and RNAi lines, PPD5 RNAi-1, -3, and -6 plants.

Surface-sterilized seeds of wild-type *A. thaliana* var. Columbia (Col-0) were germinated on solid MS medium [17] containing 2% sucrose and 0.7% agar and then incubated for 2 days at 4°C in the dark. The seedlings were transferred to soil 14 days later and grown at 20°C under 50–80 µmol photons m^−2^ s^−1^ of white light under 8 h light/16 h dark diurnal conditions. Rosette leaves of 6–8-week-old plants were used for subsequent analyses. Seeds for the T-DNA insertional mutant SALK_061118 (*ppd5*) were purchased from a collection developed at the Salk Institute Genomic Analysis Laboratory [18]. The gene structure of *PPD5* is shown in Figure 1A along with the locations of the T-DNA insertion (SALK_061118). Seeds from the *ppd5* mutant were germinated on medium supplemented with sucrose and 50 mg/l kanamycin the antibiotic-resistant seedlings were transplanted to soil pots, and homozygous individuals were confirmed using gene-specific and T-DNA-specific primers (PPD5-LP (5′-CATCTCTCATGGTTTTGGTG G-3′), PPD5-RP (5′-GATGGGGAGCCTTACTGGTAC-3′) and Lba1 (5′-TGGTTCACGTAGT GGGCCATCG-3′)). Seeds from these individuals were used for all subsequent analyses.

### RNAi Line Construction

The pHANNIBAL vector [19] was used to construct an intron-spliced hairpin RNA (RNAi construct). Because *PPD5* is a member of an extended gene family, a region of the gene unique to *PPD5* with little sequence similarity to the other *PPD* or *PPL* genes was chosen as the RNAi target. The sequence (+49 to +232) of the *PPD5* gene (At5g11450) was chosen for expression suppression (Figure 1A). This construct will be referred to as PPD5-RNAi. The primers were 5′-CCGCTCGAGATGGCTCTTCTCTGTCCTTC-3′ and 5′-GGGGTACCGACCAATGAGCA CTAAATCC-3′ for the sense fragment and 5′- GCTCTAGAATGGCTCTTCTCTGTCCTTC-3′ and 5′-CCATCGATGACCAATGAGCACTAAATCC-3′ for the antisense fragment. After construction and verification by sequencing, the expression region was excised from pHANNIBAL with NotI and then subcloned into pART27. The PPD5-RNAi plasmid was introduced into *Agrobacterium* (strain GV3101) by the freeze–thaw method [20]. Healthy, flowering WT plants were then transformed by the floral dip method as described previously [21]. Surface-sterilized seeds were spread on solid MS medium containing 2% sucrose, 0.7% agar, 50 mg/l kanamycin, and 400 mg/l carbenicillin and then incubated for 2 days at 4°C in the dark. The transgenic kanamycin-resistant seedlings were transferred to soil after the first true leaves appeared and grown as described above. The presence of the transgene in the kanamycin-resistant plant lines was confirmed by PCR using construct-specific primer pairs. More than twenty independent PPD5 RNAi lines were obtained. Individuals from lines PPD5 RNAi-1, -3, and -6 were characterized in this work.

### Expression Analysis

The relative amounts of transcripts in the different plant lines were determined by reverse transcriptase PCR (RT-PCR). Total RNA was extracted from leaves of 6-week-old plants using a SurePrep Plant/Fungi Total RNA Purification Kit (Fisher Bioreagents). cDNA was synthesized from 1 µg total RNA using M-MuLV Reverse Transcriptase and the primer dT_23_VN (New England BioLabs). The ubiquitin gene, *UBQ10*, was amplified with primers 5′-GATCTTTGCC GGAAAACAATTGGAGGATGGT-3′ and 5′-CGACTTGTCATTAGAAAGAAAGAGATAA CAGG-3′. *PPD5* transcripts were amplified with primers PPD5_5 (5′- CGCCATAGC TATGGCTCTTCTC-3′), PPD5_3A (5′- TCACCAAAACCTCCATGGATCC-3′) and PPD5_3B (5′- GGACAAGGTACTCGTAGTACCA-3′).

### Fluorescence Measurements

For all of the fluorescence experiments, leaves from wild type and *ppd5* mutants were excised and dark-incubated for 5 min before initiation of the experiments. Fluorescence induction was monitored with a Photon Systems Instruments (PSI, Czech Republic) FL3000 dual modulation kinetic fluorometer (a commercial version of the instrument described in [22]). Both measuring and saturating flashes are provided by computer-controlled photodiode arrays. The fluorescence induction curves were transformed according to (1−F_o_/F_t_)/(F_v_/F_m_) and fit to a three component exponential function [23]. The NDH activity and nonphotochemical quenching (NPQ) measurements were performed using a Joliot-Type Spectrophotometer (JTS-10, Bio-Logic Scientific Instruments) in fluorescence mode. For NDH activity measurements, samples were illuminated with actinic light (PS I excitation, 67 µmol photons m^−2^ s^−1^) for 3.5 min followed by 3 min of darkness with measuring pulses (5.7 µmol photons m^−2^ s^−1^) throughout the entire sequence. For NPQ measurements, samples were subjected to 150 ms saturating pulses (7900 µmol photons m^−2^ s^−1^) throughout a 3.5 min period of actinic light (340 µmol photons m^−2^ s^−1^) treatment. The fluorescence parameters were determined as follows: initial fluorescence of dark-adapted samples (F_o_), maximal fluorescence of dark-adapted samples (F_m_), maximal fluorescence of light-adapted samples (F′_m_), steady state fluorescence of light-adapted samples (F_s_); F_v_/F_m_ = (F_m_−F_o_)/F_m_; NPQ = (F_m_−F′_m_)/F′_m_; ΦPS II = (F′_m_−F_s_)/F′_m_ [24], [25], [26]. Data were analyzed using Origin version 8.1 and proprietary software provided by Photon Systems Instruments and Bio-Logic Scientific Instruments.

### Immunodetection

For analysis of the thylakoid membrane protein complement, thylakoid membranes were isolated from wild type and *ppd5* mutant plants as described previously [27]. Chlorophyll concentration was determined by the method of Arnon [28]. LiDS–PAGE was performed on 12.5–20% polyacrylamide gradient gels with 5 µg of chlorophyll loaded per lane. Specific antibodies were used to detect the PsaB, CP47, cytochrome *f* and Ndh-L proteins. For detection of the immobilized antibodies, the appropriate peroxidase-conjugated secondary antibodies were used followed by detection with a chemiluminescent substrate (SuperSignal WestPico chemiluminescent substrate, Pierce), and the blots were exposed to X-ray film.

### Pigment Measurements

Leaves were ground in liquid nitrogen; pigments were extracted with 80% acetone and measured spectrophotometrically on a Shimadzu UV-1800 (Shimadzu Scientific Instruments). Total chlorophyll and total carotenoid pigment composition was calculated by the method of Lichtenthaler [29]. Values are given as µg pigment per g fresh weight (f.w.).

### Root Phenotype Analysis

Wild-type and *ppd5* mutant seeds were germinated as described above. After 14 days of growth at 20°C under 50–80 µmol photons m^−2^ s^−1^ of white light under 8 h light/16 h dark diurnal conditions, seedlings were removed from the agar, mounted on slides and visualized using a dissecting microscope. For the GR24 complementation experiment, wild-type and *ppd5* mutant seeds were germinated on plates containing MS medium 2% sucrose in the presence or absence of 5 µM GR24 and grown vertically under the conditions described above. Roots were analyzed after 14 days of growth.

## Results

### Screening Transgenic Plants

The expression of the *PPD5* transcript was determined by RT-PCR in wild-type and *ppd5* mutant plants. The T-DNA mutant, *ppd5*, contained no detectable full-length *PPD5* transcript amplified from primers PPD5_5 and PPD5_3A (Figure 1B). The numerous introns and exons within the *PPD5* gene and the fact that the T-DNA insertion was located near the 3′ end of the gene raised the possibility that alternative transcripts could be present in the *ppd5* mutant, either as chimeric transcripts, truncated transcripts or alternatively spliced forms. These transcripts could give rise to truncated versions of the PPD5 protein. Indeed, transcripts truncated at the exon immediately upstream of the T-DNA insertion amplified by primers PPD5_5 and PPD5_3B were detected in the *ppd5* mutant (Figure 1B). In the three different PPD5 RNAi lines, however, no *PPD5* transcripts were detected (Figure 1B). Notably, transcripts for the other PsbP family members (*PSBP*, *PPL1-2*, and *PPD1-4*, and *6*) were detected in the PPD5 RNAi lines (data not shown), indicating that *PPD5* is specifically suppressed in these plants.

### Photosynthetic Function

**Figure 2:**
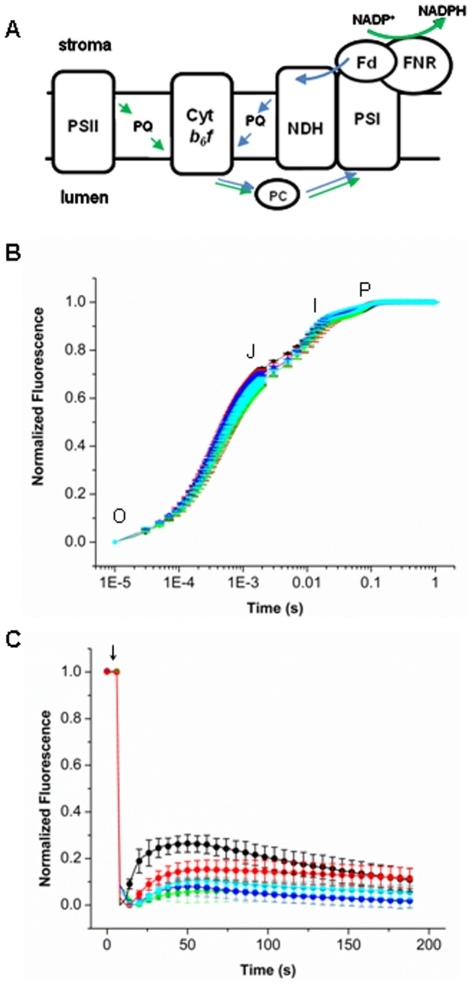
Fluorescence Measurements. (A) Schematic of photosynthetic electron transfer. PSII, photosystem II; Cyt *b_6_f*, cytochrome *b_6_f*; PC, plastocyanin; PQ, plastoquinone; NDH, NADPH Dehydrogenase; PSI, photosystem I; Fd, ferredoxin; FNR, ferredoxin:NADP^+^ reductase. Green arrows denote linear electron transfer and blue arrows denote cyclic electron transfer. (B) Fluorescence induction curves of WT (black), *ppd5* (red), PPD5 RNAi-1 (green), PPD5 RNAi-3 (blue), and PPD5 RNAi-6 (cyan). All curves were normalized according to (1-F_o_/F_t_)/(F_v_/F_m_). Error bars represent the standard deviation of n = 3–5 measurements. (C) NDH activity in WT (black), *ppd5* (red), PPD5 RNAi-1 (green), PPD5 RNAi-3 (blue), and PPD5 RNAi-6 (cyan) is shown as the transient chlorophyll fluorescence rise and decay after the actinic light has been turned off (arrow). Curves have been normalized to the maximal and minimal fluorescence values for each curve. Error bars represent the standard deviation of n = 3–5 measurements.

Since PPD5 is related to the PsbP protein functioning in PS II and is also located in the thylakoid lumen, and since many of the PsbP-like proteins have been shown to affect various aspects of photosynthesis [10], we hypothesized that *ppd5* mutants would be defective in photosynthetic electron transport. To examine linear photosynthetic electron transport we performed chlorophyll fluorescence induction experiments. Using a logarithmic timing series, a polyphasic fluorescence rise exhibits typical OJIP transients. The OJIP transients report the step-wise accumulation of closed PS II centers upon illumination. Thus, the fluorescence rise kinetics provide information on linear electron flow through PS II and also probe other components affecting the redox equilibrium of the plastoquinone (PQ) pool [23], [26]. Figure 2A shows components and electron carriers of the photosynthetic electron transfer chain. The initial O-J transition constitutes the photochemical phase of the chlorophyll *a* fluorescence rise and can be ascribed to PQ pool reduction during the period when electron transport from the PQ pool to cytochrome *b_6_f* complex is maximal. The I-P phase reflects the further oxidation of the PQ pool due to the accumulation of reduced electron carriers beyond the cytochrome *b_6_f* complex.

Figure 2B shows the chlorophyll *a* fluorescence induction curves observed in wild-type, *ppd5* and PPD5 RNAi mutant plants. The fluorescence induction curves of the wild-type, *ppd5* and PPD5 RNAi mutant plants were very similar, indicating that PS II and linear photosynthetic electron transfer were largely unaffected by the absence of the PPD5 protein. The induction curves were fit to a three component exponential function, and the amplitudes and lifetimes of the OJ, JI, and IP components are given in Table 1. There is a small increase in the JI amplitude for the PPD5 RNAi-3 and -6 plants indicating minor problems with electron transfer between PS II and the cytochrome *b_6_f* complex.

**Table 1:** Chlorophyll fluorescence induction fit and photosynthetic parameters.

	WT	*ppd5*	PPD5RNAi-1	PPD5RNAi-3	PPD5RNAi-6
**A_OJ_ (%)**	66±0	63±3	60±3	64±1	60±1
**t_OJ_ (µs)**	427±15	435±42	447±6	397±15	423±6
**A_JI_ (%)**	22±1	21±1	26±1	27±3	30±1
**t_JI_ (ms)**	7±0	7±0	7±1	8±1	7±0
**A_IP_ (%)**	11±0	16±3	14±3	9±3	10±2
**t_IP_ (ms)**	50±4	35±1	39±5	49±10	43±3
**F_o_**	0.46±0.07	0.44±0.07	0.57±0.01	0.63±0.06	0.59±0.05
**F_m_**	2.01±0.41	1.86±0.45	2.30±0.16	2.39±0.36	2.33±0.24
**F_v_/F_m_**	0.77±0.01	0.76±0.02	0.75±0.02	0.74±0.01	0.75±0.01

Error represents the standard deviation of n = 3–5 measurements.

Chlorophyll fluorescence measurements were also used to determine a number of additional photosynthetic parameters (Table 1). The F_o_, F_m_, and F_v_/F_m_ photosynthetic parameters were calculated from the raw data of the fluorescence induction curves. While the PPD5 RNAi lines had slightly higher F_o_ values relative to wild type, the F_v_/F_m_ values, representing the maximum quantum yield of photosynthesis, were equivalent among wild-type and the *ppd5* mutants. Photosynthetic performance was also assayed using pulsed amplitude modulated fluorescence to probe additional parameters, which are shown in Table 2. Again, the maximum quantum yield value, F_v_/F_m_ was the same for all samples. After actinic light treatment (340 µmol photons m^−2^ s^−1^), the ΦPS II value, a measure of actual quantum yield of PS II, was slightly lower for the PPD5 RNAi lines relative to wild type. Nonphotochemical quenching (NPQ) is the thermal energy dissipation which is a photoprotective mechanism for dealing with excess light [30]. There was a significant increase in the NPQ parameter in the *ppd5* and PPD5 RNAi mutants relative to wild type. Therefore, after light treatment in this measurement, the *ppd5* and PPD5 RNAi mutants had an increase in thermal energy dissipation and a small decrease in photochemical yield.

**Table 2:** Photosynthetic parameters from pulsed amplitude fluorescence measurement.

	WT	*ppd5*	PPD5RNAi-1	PPD5RNAi-3	PPD5RNAi-6
**F_v_/F_m_**	0.83±0.01	0.83±0.01	0.82±0.01	0.82±0.001	0.81±0.001
**ΦPS II**	0.21±0.02	0.21±0.05	0.15±0.01	0.16±0.01	0.16±0.02
**NPQ**	1.31±0.02	1.43±0.04	1.52±0.05	1.50±0.01	1.48±0.07

Error represents the standard deviation of n = 3–5 measurements.

Because previous studies have suggested the PsbP protein family members play a role in NDH function and assembly [10], NDH activity was also examined. Figure 2A illustrates the photosynthetic electron transfer chain including the contribution of the NDH complex to cyclic electron transfer between the cytochrome *b_6_f* complex and PS I. During cyclic electron transfer, the plastoquinol utilized by the cytochrome *b_6_f* complex is supplied by NDH rather than PS II. NDH activity is measured by a transient fluorescence increase after switching off actinic illumination [31]. This transient increase is due to the delivery of electrons from stromal electron carriers to the plastoquinone pool. Notably, in plants, it has recently been shown that the electron donor is ferredoxin rather than NADPH; consequently the “NDH” nomenclature has been questioned [32]. Nevertheless, this transient increase in the amount of plastoquinol slows the transfer of electrons from Q_A_^−^ to Q_B_ and leads to a transient increase in the PS II fluorescence. Figure 2C shows the NDH activity measurements of wild-type, *ppd5* and PPD5 RNAi mutant plants. In the *ppd5* and PPD5 RNAi mutant plants, the fluorescence transient was significantly smaller than that of wild-type plants. These results indicate that a modest decrease in NDH complex activity is observed in plants lacking the PPD5 protein.

**Figure 3:**
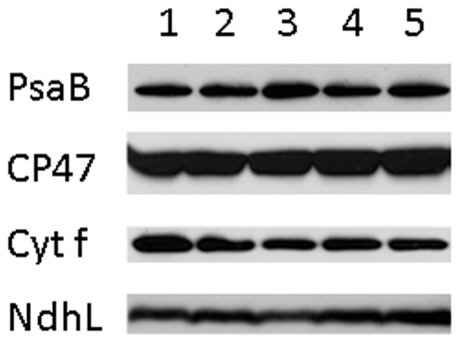
Thylakoid Protein Accumulation. Thylakoid membranes from wild-type and mutant plants probed with antibodies against the PsaB (a marker for PS I), CP47 (a marker for PS II), cytochrome *f* (a marker for the cytochrome *b_6_f* complex), and NdhL (a marker for the NDH complex) proteins. Lane 1, wild type; lane 2, *ppd5*; lane 3, PPD5 RNAi-1; lane 4, PPD5 RNAi-3; lane 5, PPD5 RNAi-6.

The photosynthetic defects evident from the above measurements for the *ppd5* and PPD5 RNAi mutants are relatively mild. The relative amounts of thylakoid membrane proteins representing the complexes of the photosynthetic electron transfer (PsaB, PS I; CP47, PS II; Cyt *f*, cytochrome *b_6_f* complex; and NdhL, NDH) in wild type, *ppd5* and PPD5 RNAi mutant are shown in Figure 3. Normal levels of all of these marker proteins were found in the *ppd5* and PPD5 RNAi mutants. Therefore, despite a significant decrease in NDH activity there is normal accumulation of the complex in the thylakoids of these mutants.

### Plant Morphology

**Figure 4:**
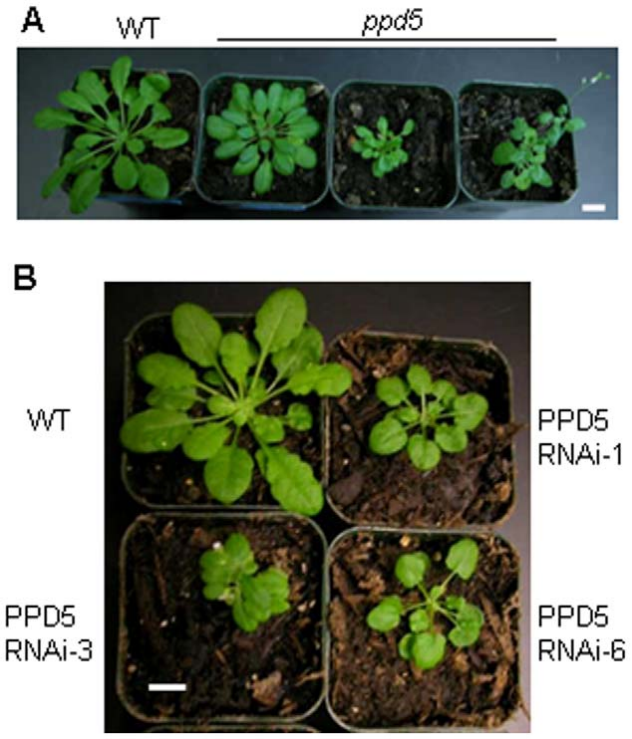
Comparison of wild-type and mutant plants. (A) 7-week-old WT and *ppd5* T-DNA plants. (B) 7-week-old WT, PPD5 RNAi-1, -3, and -6 plants. Scale bars = 1 cm.

**Figure 5:**
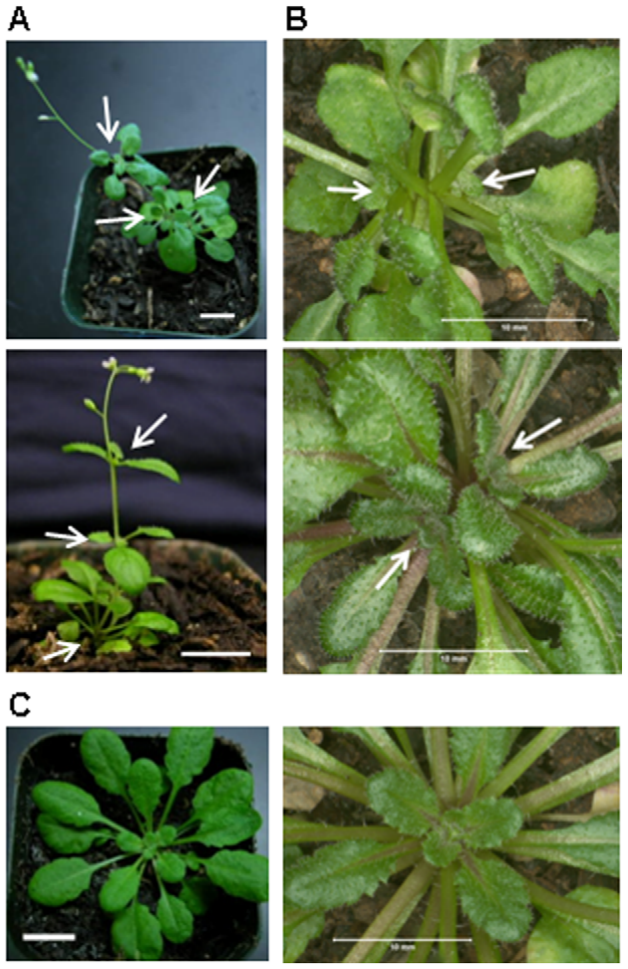
Aberrant morphology in *ppd5* and PPD5 RNAi plants. (A) Individuals displaying the aerial rosette phenotype. The aerial rosettes are indicated with white arrows. (B) Individuals displaying the multiple rosette phenotype. The rosettes are indicated with white arrows. (C) Wild-type individuals shown in comparable views of A and B. Scale bars = 1 cm.

**Figure 6:**
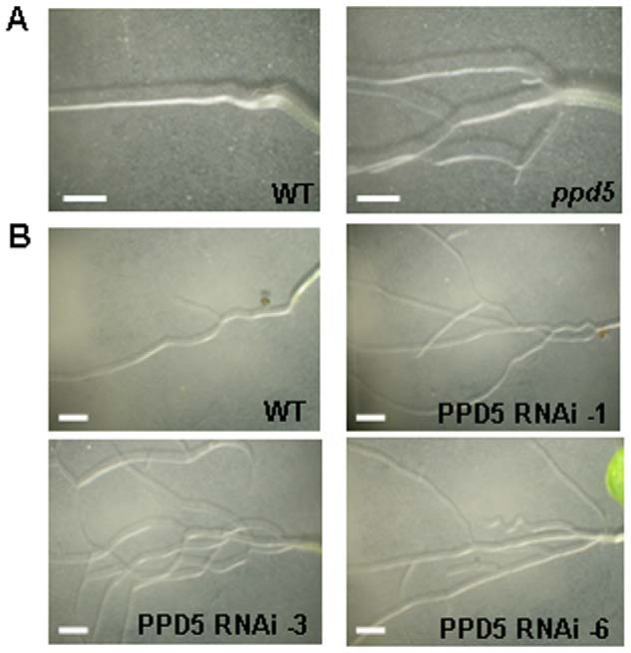
Root-branching phenotype. (A) Root structures of 14-day-old WT and *ppd5* individuals. (B) Root structures of 14-day-old WT, PPD5 RNAi-1, -3, and -6 individuals. Scale bars = 2 mm.

The *ppd5* and PPD5 RNAi plants were typically smaller than wild-type plants and frequently displayed morphological defects (Figure 4). The two main types of aberrant shoot morphology observed were aerial rosettes (Figure 5A) and multiple rosettes (Figure 5B). In the aerial rosette phenotype, additional rosettes formed at the axils of the first few nodes of the growing stem. In the multiple rosette phenotype, axillary buds developed into new rosettes in the axils at the base of the plant. Therefore, the *ppd5* and PPD5 RNAi plants with these morphological abnormalities contained multiple rosettes, either within the basal rosette or along the growing stem. The *ppd5* and PPD5 RNAi plants also displayed altered root architecture relative to wild-type plants (Figure 6). The root morphology of the mutants was strikingly different from that observed for wild-type plants. The 14-day-old wild-type seedlings exhibited very few lateral roots in the vicinity of the root-shoot transition zone, but the *ppd5* and PPD5 RNAi mutant individuals often had multiple lateral roots initiating at the root-shoot junction. It should be noted that the aberrant root architecture was observed in the majority (>90%) of *ppd5* and PPD5 RNAi seedlings analyzed (72 individuals), whereas the defects in shoot morphology were observed in only ∼25% of plants from the different *ppd5* and PPD5 RNAi lines (60 individuals).

However, similar root- and shoot-branching phenotypes have also been reported for mutants defective in strigolactone synthesis or signaling [33], [34], [35], [36], [37], [38], [39]. Strigolactones are carotenoid-derived plant hormones that negatively regulate axillary bud development [40], [41], [42], [43]. Thus, mutants defective in strigolactone synthesis display increased branching phenotypes and have recently been shown to have altered root architecture [33], [36], [37], [38], [39]. The strigolactone biosynthetic pathway has not been fully elucidated; however, all of the biosynthetic enzymes identified to date are localized in the chloroplast stroma [33], [36], [44], [45]. Because the PPD5 protein is also chloroplast-localized, it may be involved in the strigolactone biosynthesis pathway.

**Figure 7:**
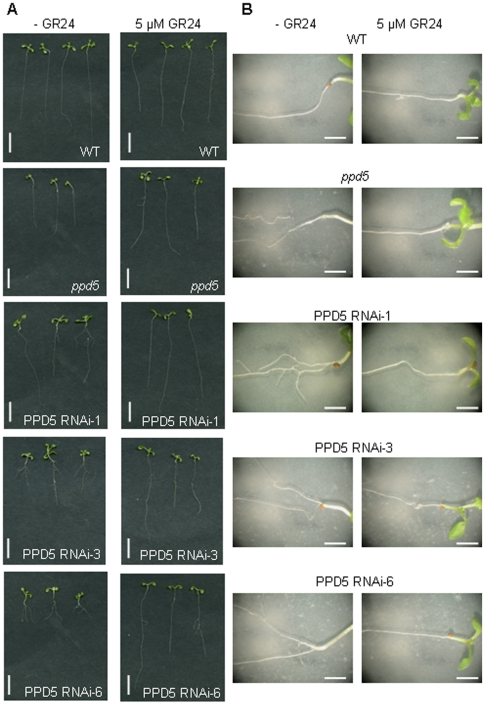
GR24 complementation of root-branching phenotype. (A) 14-day-old seedlings of WT, *ppd5* and PPD5 RNAi-1, -3, and -6 grown in the absence and presence of 5 µM GR24. Scale bar = 1 cm. (B) Root structures of WT, *ppd5* and PPD5 RNAi-1, -3, and -6 grown in the absence and presence of 5 µM GR24. Scale bar = 2 mm.

To test the hypothesis that the morphological defects observed are due to a strigolactone deficiency in the *ppd5* and PPD5 RNAi plants, mutant plants were germinated in the presence and absence of the synthetic strigolactone GR24 to chemically complement the root-branching phenotype. The results are shown in Figure 7. In the absence of GR24, the *ppd5* and PPD5 RNAi individuals displayed increased root branching relative to wild-type plants. However, the presence of 5 µM GR24 in the medium chemically complemented the root phenotype observed in the mutants such that they exhibited longer primary roots and the absence of multiple lateral roots originating at the root-shoot junction. This result strongly supports the hypothesis that the morphological defects observed in the *ppd5* and PPD5 RNAi lines are related to a strigolactone deficiency. Notably, there were no significant differences in the total chlorophyll and total carotenoid contents among wild-type, *ppd5*, and PPD5 RNAi plants (Table 3). Therefore, the strigolactone deficiency in the *ppd5* mutants is likely to be further downstream from the carotenoid precursors detectable in the visible spectrum.

**Table 3:** Pigment content of plants.

	WT	*ppd5*	PPD5RNAi-1	PPD5RNAi-3	PPD5RNAi-6
**Chlorophyll** **µg/g f.w.**	1330±83	1300±101	1478±170	1329±191	1406±198
**Carotenoids** **µg/g f.w.**	257±15	231±19	266±25	235±33	241±32

Error represents the standard deviation of n = 10–12 measurements. (f.w. = fresh weight).

## Discussion

The members of the PsbP family of proteins examined previously have been shown to exhibit a variety of functions, all of which are related to electron transfer reactions within the thylakoid membranes. In the case of the PPD5 protein, only modest effects on photosynthetic electron transport were observed. Indeed, under the conditions analyzed, only mild defects in linear electron transfer were apparent for either the *ppd5* or PPD5 RNAi mutants. NDH activity was reduced somewhat in these mutants, suggesting a diminished capacity for cyclic electron transfer around PS I via NDH and the cytochrome *b_6_f* complex. While the cyclic electron transfer mediated by NDH complexes is important for preventing the over-reduction of stromal electron acceptors, its significance under normal growth conditions is likely to be small [46], [47]. It has been suggested that NDH activity is important for adapting the photosynthetic machinery to stress conditions such as heat, light or drought when the functioning of linear electron transfer results in redox imbalances of the various electron acceptor pools [1]. These correlations have largely been based on upregulation of NDH subunits under stress conditions, with only small differences in photosynthetic parameters for *ndh* mutants under various stress conditions, ranging from decreases, no change, and increases in NPQ induction [1], [48], [49], [50].

The most striking differences between the wild-type and mutant plants were the observed morphological defects, in which the *ppd5* and PPD5 RNAi mutants displayed both an increase in lateral root formation and the development of additional rosettes from their axils both in the basal rosette and along the elongating stem. Importantly, no plant morphology phenotypes have been reported for the numerous other NDH subunit mutants, which display no electron transport activity or complex accumulation [1]. Interestingly, available expression data indicate that *PPD5* is most highly expressed in leaf primordia, cotyledons, juvenile leaves, axillary buds, and axillary shoots [51]. Therefore, defects in normal axillary growth are consistent with the expression profile of *PPD5*. If this is the case, PPD5 would be the first thylakoid lumenal protein identified to participate in this process. It should be noted that it is formally possible that the PPD5 protein may also be localized to other subcellular compartments besides the chloroplast lumen; however this has not been experimentally evaluated.

Interestingly, strigolactones have also been linked to photosynthetic processes on the basis of transcript profiling in tomato [52]. Treatment with the synthetic strigolactone GR24 led to the induction of a number of genes encoding subunits of the photosynthetic electron transfer chain, including PS I, PS II, cytochrome *b_6_f*, and light-harvesting components. Notably, among the genes upregulated (>23×) in response to GR24 were also subunits of the NDH complex (NdhD and NdhH), ferredoxin I, and the minor light-harvesting protein Lhca6. It has been recently shown that the chloroplast NDH complex accepts electrons from ferredoxin [32], and the formation of NDH-PS I supercomplexes necessary for efficient cyclic electron transfer is mediated by Lhca6 [53], [54]. These results suggest that the capacity for cyclic electron transfer is upregulated in response to strigolactone treatment, while the data presented in this work provide evidence that a decrease in cyclic electron transfer capacity is correlated with strigolactone deficiency.

It must be stressed that our understanding of the strigolactone biosynthetic pathway is far from complete, and the point at which it diverges from established carotenoid biosynthetic pathways is unknown. Interestingly, the activities of many carotenoid biosynthetic enzymes appear to be redox-controlled. This is true for the desaturases (phytoene desaturase and ζ-carotene desaturase), whose reactions require net electron transfer, as well as other enzymes that do not require electron transfer, including carotenoid isomerase and lycopene cyclases [55], [56], [57]. The reduced NDH activity observed in the *ppd5* and PPD5 RNAi mutants may also have an effect on the carotenoid or strigolactone biosynthetic pathways. Recently, a study of the tomato *Orange Ripening* (*ORR^Ds^*) mutant in tomato has correlated the loss of the NdhM subunit with a decrease in carotenoid accumulation during fruit ripening [56]. A previous report has even linked the NAD(P)H:quinone oxidoreductase activity necessary for phytoene desaturase activity in *Narcissus* chromoplasts to a protein with a high degree of sequence similarity to PsbP [55]. Given that the PsbP and PPL2 proteins have been shown to affect accumulation and tune the activities of redox enzymes in the thylakoid membrane [2], [10], it is tempting to speculate that the PPD5 protein may have an analogous function with respect to the strigolactone biosynthetic pathway. Clearly, additional experiments are necessary to elucidate any functional relationships of the PPD5 protein, photosynthetic electron transfer and carotenoid and/or strigolactone biosynthesis.

Finally, this work highlights the fact that lumenal proteins have functions beyond those normally associated with photosynthetic electron transfer. While the most extensively characterized lumenal proteins have been shown to function in the assembly and regulation of the photosynthetic apparatus, numerous other lumenal constituents (including immunophilins, proteases, peroxidases and pentapeptide repeat proteins) have been identified in proteomic studies [15]. It is likely that at least some of these components function in processes other than photosynthetic electron transport.
